# Non-invasive Brain and Spinal Stimulation for Pain and Related Symptoms in Multiple Sclerosis: A Systematic Review

**DOI:** 10.3389/fnins.2020.547069

**Published:** 2020-11-20

**Authors:** Chiara Zucchella, Elisa Mantovani, Roberto De Icco, Cristina Tassorelli, Giorgio Sandrini, Stefano Tamburin

**Affiliations:** ^1^Section of Neurology, Department of Neurosciences, Verona University Hospital, Verona, Italy; ^2^Department of Neurosciences, Biomedicine and Movement Sciences, University of Verona, Verona, Italy; ^3^Neurorehabilitation Unit, IRCCS Mondino Foundation, Pavia, Italy; ^4^Department of Brain and Behavioral Sciences, University of Pavia, Pavia, Italy

**Keywords:** depression, fatigue, multiple sclerosis, non-invasive brain stimulation (NIBS), non-invasive spinal stimulation, pain, systematic review

## Abstract

**Background:** Neuropathic and nociceptive pain frequently affect patients with multiple sclerosis (MS), with a prevalence close to 90% and significant impact on general health and quality of life. Pharmacological strategies are widely used to treat pain in MS, but their effectiveness and side-effects are controversial. Among non-pharmacological treatments for pain, non-invasive brain and spinal stimulation (NIBSS) has shown promising preliminary results in MS.

**Objective:** Systematic review to investigate the effect of NIBSS for the management of pain in MS.

**Methods:** A literature search using Pubmed, Science Direct and Web of Science was conducted from databases inception to February 21, 2020 for studies assessing the analgesic effect of NIBSS on pain in MS.

**Results:** A total of 279 records were title- and abstract-screened, nine were assessed for full text and included. The NIBSS techniques explored were transcranial direct current stimulation (*N* = 5), transcranial magnetic stimulation (*N* = 2), transcranial random noise stimulation (*N* =1), transcutaneous spinal direct current stimulation (*N* = 1). The targets were the primary motor cortex (M1; *N* = 4), the left dorsolateral pre-frontal cortex (DLPFC; *N* = 3), the spinal cord (*N* = 1), unspecified brain target (*N* = 1). The study designs were randomized (*N* = 7), open label (*N* = 1), single case report (*N* = 1). Despite the differences in study design, target and NIBSS technique that impeded a meta-analysis, all the studies converge in showing a significant improvement of pain after active NIBSS with less consistent effects on other symptoms of the pain-related cluster (depression, fatigue, cognition) and quality of life.

**Conclusions:** Excitatory NIBSS over M1, left DLPFC and spinal cord appear to be the most effective protocols for pain in MS. Open questions include the use of neurophysiological or neuroimaging surrogate outcome measures, the stratification of patients according to the clinical profiles and underlying pathogenetic mechanisms and the combination of NIBSS to pharmacological treatment, neurorehabilitation, or psychotherapy to improve the clinical effect. The duration of the effect to NIBSS and the feasibility and efficacy of telemedicine NIBSS protocols are other open key questions.

## Introduction

Pain is common in patients with multiple sclerosis (MS; O'Connor et al., 2008) and has a significant burden on general and psychological health, quality of life (QoL), work and social role (Kalia and O'Connor, 2005; Foley et al., 2013). Pain was reported in 29–86% of MS patients, such a wide range being due to different diagnostic criteria and assessment methods, heterogeneity of the samples, and different disease stages span when pain was assessed (Nurmikko et al., 2010; Thompson et al., 2010; Foley et al., 2013; Solaro et al., 2013).

MS-related pain may present with high variability in terms of clinical presentation, severity, onset (Feinstein et al., 2015) and may be reported at any stage of the disease including the early ones (Solaro et al., 2004), but its prevalence increases over time because of the disease process itself, MS-related complications and aging (Khan et al., 2013).

The most common types of pain in MS are classified as nociceptive or neuropathic pain (Magrinelli et al., 2013). Nociceptive pain is a physiological response secondary to the activation of nociceptors aimed to warn the brain of real or potential tissue damage. In contrast, neuropathic pain is due to a lesion or disease of the peripheral or central parts of the somatosensory system (Finnerup et al., 2016). Pain associated with MS stands amongst the most common causes of chronic neuropathic pain (Scholz et al., 2019).

Nociceptive pain in MS patients includes (a) musculoskeletal system, which is often related to abnormal or forced posture, (b) headache, which may predate or be unrelated to MS, (c) post-traumatic pain, and (d) pain secondary to treatment, e.g., painful pathological fractures secondary to long-term steroid use and immobilization causing osteoporosis (Solaro et al., 2018). MS patients may experience both pain and spasticity, and pain secondary to spasticity or painful tonic spasms is a subtype of nociceptive pain frequently reported in MS (Solaro et al., 2018). Among primary headaches, migraine was reported to be three times more frequent in MS patients than in the general population, to carry a considerable disability, and to be associated with a more symptomatic course and an increased contrast enhancing lesion activity compared to MS patients without headache (Kister et al., 2010; Graziano et al., 2015).

Central neuropathic pain in MS includes (a) ongoing neuropathic pain of limbs, (b) Lhermitte's phenomenon, (c) trigeminal neuralgia, and (d) pain associated with optic neuritis, all of which are associated with inflammation and secondary degeneration of central nervous system sensory pathways (Truini et al., 2013; Solaro et al., 2018).

MS patients may also report, to a variable extent, psychogenic, idiopathic or mixed pain (Truini et al., 2013). Psychogenic pain is defined as somatoform pain associated with psychiatric conditions (i.e., depression, or anxiety), or as pain behaviors associated with chronic refractory pain; idiopathic pain includes poorly understood and to some extent controversial chronic pain conditions, such as fibromyalgia, or persistent idiopathic facial pain, while mixed pain includes different pain types, often difficult to separate and quantify, caused by the same disease through different pathophysiological mechanisms (Truini et al., 2013).

According to the neuropathic pain definition and grading system (Finnerup et al., 2016), neuropathic pain can be separated from nociceptive and other types of pain based on the clinical or instrumental demonstration of a lesion or disease involving the somatosensory system (La Cesa et al., 2015; Porro et al., 2016), but this task may be difficult in MS patients, because of the frequent clinical or subclinical involvement of posterior columns of the spinal cord and/or brain somatosensory pathways.

Pain can interfere with daily functioning by reducing mobility, working activities, and engagement in recreational activities, and may cause a consistent impairment of participation in home, social, and other activities (Ehde et al., 2003; Svendsen et al., 2003, 2005; Kalia and O'Connor, 2005; Grasso et al., 2008; Gromisch et al., 2019). MS is one of the most common causes of neurological disability in young adults, and MS-related pain may impact this population of working-age patients and represent an independent risk factor for social disadvantage (Shahrbanian et al., 2013).

Pain is frequently associated to fatigue, depression and cognitive complaints in MS patients (Penner et al., 2007; Trojan et al., 2007), and these three symptoms may influence each other (Harrison et al., 2015; Marck et al., 2017), being considered a symptom cluster with some shared pathogenetic mechanisms and that should be targeted together to improve patients' QoL (Shahrbanian et al., 2015).

Despite its high prevalence and severe burden, MS-related pain is still an ongoing and challenging issue with no established treatment. Pharmacological treatment of pain in MS patients is based on guidelines derived from other clinical conditions (Finnerup et al., 2015) and includes (a) non-steroidal anti-inflammatory drugs for nociceptive pain, (b) anticonvulsant, (c) antidepressants, and (d) botulinum toxin for neuropathic pain, (e) cannabinoids, (f) muscle relaxants and (g) intrathecally administered baclofen for pain secondary to spasticity or to painful tonic spasms, (h) opioid analgesics for mixed pain (Solaro et al., 2013). However, results of pharmacological approaches, even with complex therapeutic schemes, are often poor and disturbing side effects, such in the case of opioids, frequently cause the patients to drop-out (Urits et al., 2019).

The need of more effective treatments with safer profiles and fewer adverse effects has paved the way to non-pharmacological interventions for pain in MS (Amatya et al., 2018; Aboud and Schuster, 2019). In this field, evidence on neuromodulation through non-invasive brain and spinal stimulation (NIBSS) has been published in recent years, and preliminary results appear to be promising (Abboud et al., 2017; Iodice et al., 2017). NIBBS techniques can be grouped into two categories, namely electrical and magnetic stimulation, according to the differential way of inducing their neurobiological effects. Electrical stimulation is the application of current/voltage to two or more surface electrodes, whereas magnetic stimulation results from a current passing through a coil positioned on the head to generate a magnetic field, inducing in turn an electrical field and a current density field in the brain (Peterchev et al., 2012). Electrical stimulation techniques include, but are not limited to, transcranial direct current stimulation (tDCS), transcranial alternating current stimulation, transcranial random noise stimulation (tRNS), and transcutaneous spinal direct current stimulation (tsDCS).

tDCS and repetitive transcranial magnetic stimulation (rTMS) are the most widely used types of non-invasive neuromodulation techniques.

tDCS is based on a battery-powered device connected to two electrodes that deliver low-amplitude direct currents that induce neuronal membrane depolarization or hyperpolarization leading to changes in the excitability of specific brain areas being stimulated (Nitsche and Paulus, 2000). In healthy subjects, anodal tDCS delivered to the motor cortex causes neurons depolarization and increased cortical excitability, while cathodal tDCS hyperpolarizes neurons, thus reducing cortical excitability (Nitsche and Paulus, 2000). However, the effect of tDCS polarity on motor cortex excitability might not be generalized to other cortices, and several factors (e.g., stimulation duration, current intensity, tDCS setup) may affect the direction of the induced effects (Lefaucheur et al., 2017).

tDCS induces sustained changes in cortical excitability if applied for a sufficient period of time, (Nitsche and Paulus, 2001). tDCS is safe and has been reported to cause only mild side effects, e.g., skin irritation or burning sensation, especially when used daily and/or with higher current intensity (Antal et al., 2017). This side effect could be minimized when using saline-soaked electrodes (Antal et al., 2017).

rTMS is delivered to the brain by a phasic electrical current that circulates through an insulated wire coil placed over the skull and generates a transient high-intensity magnetic field, which propagates in space and induces a secondary current that depolarizes neurons in targeted brain regions, finally leading to neuroplastic changes (Paulus et al., 2013). High- and low-frequency rTMS have short-lasting excitatory and inhibitory effects on the motor cortex, respectively (Paulus et al., 2013), but the effect of rTMS frequency cannot be generalized to all cortical sites (Lefaucheur et al., 2020). Theta-burst stimulation (TBS) is a novel modified rTMS technique that causes consistent, long-lasting facilitatory and inhibitory effects on synaptic transmission according to the TBS protocol used (Huang et al., 2005). Intermittent TBS (iTBS) causes prevalent facilitation, while continuous TBS leads to prevalent inhibition (Huang et al., 2005). rTMS side effects are transient and include headache, scalp discomfort and hearing disorders, more commonly after high-frequency treatments, while epileptic seizures very seldom occur if appropriate guidelines are applied and patients are accurately selected (Rossi et al., 2009; Lefaucheur et al., 2020).

New NIBSS protocol have been recently introduced in the clinical setting, including tRNS (Terney et al., 2008) and tsDCS (Berra et al., 2019).

tRNS is a non-invasive transcranial electrical stimulation technique that produces a random electrical oscillation spectrum within defined thresholds, following the Gaussian curve around an offset midpoint (Terney et al., 2008). tRNS was reported to induce consistent excitability increases lasting 60 min after stimulation when applied to the primary motor cortex (M1), with higher frequencies (100–640 Hz) being responsible for generating this hyperexcitability probably through repeated opening of sodium channels. tRNS was found to have similar effects than tDCS without the constraint of current flow direction sensitivity characteristic of the latter (Terney et al., 2008; Palm et al., 2016).

Anodal tsDCS may represent a potentially self-administered NIBSS technique in those clinical conditions that are characterized by changes in spinal cord interneurons, and was found to inhibit nociceptive specific responses, such as the nociceptive withdrawal reflex (NWR; Cogiamanian et al., 2011) and the NWR temporal summation threshold (TST; Perrotta et al., 2016), which may contribute to the pathogenesis of pain in MS (Berra et al., 2019).

Very recent evidence-based guidelines indicated level A evidence (definite efficacy) for high-frequency rTMS of M1 contralateral to the painful side for neuropathic pain and level B evidence (probable efficacy) for high-frequency rTMS of the left M1 or DLPFC for improving quality of life or pain, respectively, in fibromyalgia (Lefaucheur et al., 2020). Despite the evidence of the efficacy of high frequency rTMS for the treatment of some types of pain, NIBSS is not routinely used in patients with MS and pain. The aim of this systematic review is to collect and report data on the role of NIBSS for the management of MS-related pain.

## Methods

This systematic review was conducted according to the Preferred Reporting Items for Systematic Reviews and Meta-Analyses (PRISMA) recommendations (Liberati et al., 2009; Moher et al., 2015).

### Eligibility Criteria

Studies assessing the effect of NIBSS on MS-related neuropathic and/or nociceptive pain as primary or secondary outcome were considered eligible for this systematic review. Both controlled and exploratory studies were eligible and included and no restrictions were placed on the publication date of the studies.

We excluded reviews, commentaries, abstracts, conference papers, and studies on animal models or healthy subjects. Studies exploring NIBSS without therapeutic goals, e.g., aiming to assess neurophysiological measures to explore MS-related pathophysiology were also excluded. We also excluded studies that explored the effect of NIBSS on other MS outcomes (e.g., fatigue, motor function, spasticity, sensory function, bladder function, cognition) but did not include pain.

Outcomes of interest were pain measured with clinically validated tools (e.g., Visual Analog Scale, Numerical Rating Scale, Short Form McGill Pain Questionnaire; Brief Pain Inventory) and other MS symptoms related or secondary to pain (e.g., fatigue, spasticity, psychosocial outcomes, QoL).

According to the PICOS model, the Participants were MS patients, the Intervention was NIBSS, the Comparison was sham NIBSS, other pain treatment or no treatment, the Outcome was pain either neuropathic or nociceptive, the Study design was controlled and exploratory studies.

### Search Strategy

The Pubmed, Science Direct and Web of Science databases were searched for peer-reviewed papers exploring the therapeutic role of NIBSS for MS-related pain, published from database inception until February 21, 2020. Only studies written in English were considered. Different search strings were used according to the maximum number of Boolean operators that are allowed in each of the selected databases.

The search string for Pubmed and Web of Science was: (pain OR chronic pain OR pain management OR pain intractable OR pain measurement OR pain threshold OR nociceptors OR neuropathic pain OR neuralgia) AND (multiple sclerosis OR demyelinating disease) AND (transcranial magnetic stimulation OR TMS OR r-TMS OR theta burst stimulation OR theta burst OR TBS OR c-TBS OR i-TBS OR NIBS OR non-invasive brain stimulation OR brain stimulation OR transcranial direct current stimulation OR tDCS OR tES OR transcranial electrical stimulation OR tCS OR transcranial current stimulation).

The search strategy for Science Direct database included: (pain OR nociceptors OR neuralgia) AND (multiple sclerosis OR demyelinating disease) AND (transcranial magnetic stimulation OR TMS OR r-TMS OR theta burst stimulation), then (pain OR nociceptors OR neuralgia) AND (multiple sclerosis OR demyelinating disease) AND (theta burst OR TBS OR c-TBS OR i-TBS), (pain OR nociceptors OR neuralgia) AND (multiple sclerosis OR demyelinating disease) AND (NIBS OR non-invasive brain stimulation OR brain stimulation OR transcranial direct current stimulation), and (pain OR nociceptors OR neuralgia) AND (multiple sclerosis OR demyelinating disease) AND (tDCS OR tES OR transcranial electrical stimulation OR transcranial current stimulation).

### Study Selection

Search results were uploaded to Rayyan software, a web-based app to facilitate collaborations among reviewers during the study selection phase (Ouzzani et al., 2016). Two authors (CZ, EM) independently screened titles and abstracts. The reference lists of relevant papers were manually checked to identify additional significant studies potentially missed in the databases search. Any disagreement was planned to be solved by consensus or consulting a third reviewer (ST).

### Data Collection Procedure

Two authors (CZ, EM) independently extracted the following data from the included papers: study design (i.e., randomized, cross-over, parallel, open label, single arm trials), type of MS, sample size, gender and age of included patients, type of pain targeted by the NIBSS intervention, type of NIBSS applied, targeted central nervous system area, NIBSS protocol features, primary and secondary outcome measures, follow-up duration, side effects, and results.

### Risk of Bias Assessment

Risk of bias was assessed independently by two authors (CZ and EM) using the revised tool for Risk of Bias in randomized trials (RoB 2.0), which consists of five domains and an overall judgement (Sterne et al., 2019). The five domains are: (1) bias arising from the randomization process; (2) bias due to deviations from the intended interventions; (3) bias due to missing outcome data; (4) bias in measurement of the outcome; (5) bias in selection of the reported result (Sterne et al., 2019).

Any disagreement was planned to be solved via consensus or by consulting a third author (ST). Risk of bias was classified as “low,” “some concerns” “high.”

### Data Analysis

A systematic and descriptive analysis of the results was provided with information presented in the text and Tables 1–3 to summarize and explain the characteristics and findings of the included studies. A meta-analysis was not feasible due to the small number of studies and subjects, as well as to the methodological, clinical and statistical heterogeneity of the included studies.

**Table 1:** Overview of the tDCS studies included in the review.

**Study**		**Patients features**			**tDCS features**		**Findings**				
**Ref**	**Design**	**MS type**	**Sample size (gender, age)**	**Pain type**	**Target**	**Protocol**	**Primary outcomes**	**Secondary outcomes**	**Follow up**	**Side effects**	**Results**
Mori et al. (2010)	Randomized, parallel, double-blind sham-controlled	RR	19 (W: 11, M: 8; age 44.8 ± 27.5)	Central NP	M1	Five daily sessions, 2 mA, 20 min; anode: C3/C4, cathode: contralateral supraorbital area	Pain VAS, PMQ-SF	QoL (MSQOL-54), depression (BDI), anxiety (VAS)	4 weeks	None	Pain and QoL significantly improved to active tDCS; effects lasted 3 weeks
Ayache et al. (2016)	Randomized, double-blind, cross-over sham-controlled	RR: 11, SP: 4, PP: 1	16 (W: 13, M: 3; age 48.9 ± 10.0)	NP	Left DLPFC	Three daily sessions, 2 mA, 20 min; active- sham 3 weeks washout; anode: F3, cathode: right supraorbital region	Pain VAS, BPI	Mood (HADS), attention (ANT), fatigue (MFIS)	None	Insomnia, nausea, headache (both arms), phosphene (sham)	Pain improved to active tDCS; no effect on secondary outcomes
Kasschau et al. (2016)	Feasibility pilot	SP: 12, RR: 6, PP: 2 (EDSS: 1–8)	20 (W: 17, M: 3; age 51 ± 9.25)	NS	Left DLPFC	Ten remotely supervised tDCS sessions, 20 min and cognitive rehabilitation; uniform bilateral DLPFC (left anodal) montage	Completion of at least 8 tDCS sessions	Pain (PROMIS, VAS), fatigue (PROMIS, VAS), affect (PANAS), cognitive speed (ANT-I)	None	None relevant	Nineteen patients completed 8 tDCS sessions; all outcomes consistently improved
Rudroff et al. (2019)	Case report	RR	1 (M, 52 years)	NP	Left M1	Five daily sessions, 2 mA, 20 min; anode: C3, cathode: contralateral supraorbital area	Pain VAS, NPSI	[^18^F]-FDG PET	None	None	Pain improved and [^18^F]-FDG PET uptake increased in the thalamus after tDCS
Workman et al. (2020)	Randomized, double–blind sham-controlled cross-over pilot	RR	6 (W: 3, M: 3; age 46.7 ± 14.1)	NS	M1	Five daily sessions, 2 mA, 20 min; anode: C3/C4, cathode: contralateral supraorbital area	Pain VAS, fatigue (FSS), depression (BDI)	Isokinetic leg strength, fatigability testing	None	None	Pain, fatigability and fatigue improved to active tDCS

*ANT, Attention Network Test; ANT-I, Attention Network Test-Interaction; BDI, Beck Depression Inventory; BPI, Brief Pain Inventory; DLPFC, dorsolateral pre-frontal cortex; EDSS, Expanded Disability Status Scale; [^18^F] FDG-PET, [^18^F] fluorodeoxyglucose positron emission tomography; FSS, Fatigue Severity Scale; HADS, 14-item Hospital Anxiety and Depression Scale; M, men; mA, milliampere; MFIS, Multidimensional Fatigue Inventory Score; MPQ-SF, McGill pain questionnaire short form; MS, multiple sclerosis; MSQOL-54, Multiple Sclerosis Quality of Life-54 scale; M1, primary motor cortex; NP, neuropathic pain; NPSI, Neuropathic Pain Symptom Inventory; NS, not specified; PANAS, Positive and Negative Affect Schedule; PP, primary progressive; PROMIS, Patient Reported Outcomes Measurement Information System; QoL, quality of life; Ref, reference; RR, relapsing remitting; SP, secondary progressive; tDCS, transcranial direct current stimulation; VAS, Visual Analog Scale; W, women*.

**Table 2:** Overview of the rTMS studies included in the review.

**Study**		**Patients features**			**rTMS features**		**Findings**				
**Ref**	**Design**	**MS type**	**Sample size (gender, age)**	**Pain type**	**Target**	**Protocol**	**Primary outcomes**	**Secondary outcomes**	**Follow up**	**Side effects**	**Results**
Seada et al. (2013)	Randomized, parallel (control group: LLT)	NS	30 (age 56.4 ± 6.6)	TN	NS	10 Hz, 50 mA, 20 min	Pain NRS	Oral mouth opening, masseter and temporalis muscle tension and CMAP	None	NS	Both groups improved, no statistical comparison between the two groups
Korzhova et al. (2019)	Randomized, parallel, single blind sham-controlled	SP	34 (W: 20, M: 14)	Spasticity pain	M1	Ten sessions for 5 days for 2 weeks; HF rTMS (20 Hz, 30 min); iTBS (35 Hz, 1,200 pulses, 10 min)	Spasticity (MAS, NAS, SESS)	Pain, fatigue (MFIS)	2 and 12 weeks	None	MAS significantly improved to HF rTMS and iTBS; SESS significantly improved to iTBS and lasted at follow-up; pain and fatigue significantly improved to HF rTMS

*CMAP, compound muscle action potential; HF, high frequency; Hz, hertz; iTBS, intermittent theta-burst stimulation; LLT, low-level laser therapy; M, men; mA, milliampere; MAS, Modified Ashworth Scale; MFIS, Multidimensional Fatigue Inventory Score; MS, multiple sclerosis; M1, primary motor cortex; NAS, numerical analog scale; NRS, numerical rating scale; NS, not specified; Ref, reference; rTMS, repetitive transcranial magnetic stimulation; SESS, Subjective Evaluating Spasticity Scale; SP, secondary progressive; tDCS, transcranial direct current stimulation; TNP, trigeminal neuropathy; W, women*.

**Table 3:** Overview of the other NIBSS studies included in the review.

**Study**		**Patients features**			**NIBSS features**		**Findings**				
**Ref**	**Design**	**MS type**	**Sample size (gender, age)**	**Pain type**	**Target**	**NIBSS type and protocol**	**Primary outcomes**	**Secondary outcomes**	**Follow up**	**Side effects**	**Results**
Palm et al. (2016)	Randomized, double-blind, sham-controlled cross-over	RR: 11, SP: 4, PP: 1	16 (W: 3, M: 13; age 47.4 ± 8.9)	NP	Left DLPFC	tRNS: 3 consecutive days, 2 mA, 20–30 min; 3 weeks	Pain VAS, BPI; attention (ANT)	Depression and anxiety (HADS); fatigue (MFIS); PREPS; frontal midline theta	None	None	Pain scores and PREPS N2-P2 amplitude decreased to real tRNS
Berra et al. (2019)	Pilot randomized, parallel, double-blind sham-controlled	SP: 24, PP: 5, RR: 4 (EDSS: 5.9 ± 1.3)	33 (W: 25, M: 8; age: real 57.6 ± 9.1, sham: 54.0 ± 7.8)	NP	Spinal cord	tsDCS: 10 sessions in 2 weeks, 2 mA, 20 min; anode: thoracic spinal cord; cathode: right shoulder (suprascapular region)	NPSI, spasticity (AS), fatigue (FSS)	NWR, NWR TST	1 month	None	NPSI significantly reduced to real tsDCS with effect lasting up to 1 month; trend toward inhibition of NWR responses to real tsDCS

*ANT, Attention Network Test; AS, Ashworth scale; BPI, Brief Pain Inventory; DLPFC, dorsolateral pre-frontal cortex; EDSS, Expanded Disability Status Scale; FSS, Fatigue Severity Scale; HADS, 14-item Hospital Anxiety and Depression Scale; M, men; mA, milliampere; MFIS, Multidimensional Fatigue Inventory Score; MS, multiple sclerosis; NIBSS, non-invasive brain and spinal stimulation; NP, neuropathic pain; NPSI, Neuropathic Pain Symptom Inventory; NWR, nociceptive withdrawal reflex; PP, primary progressive; PREPS, pain related evoked potentials; Ref, reference; RR, relapsing remitting; SP, secondary progressive; tRNS, transcranial random noise stimulation; tsDCS, transcutaneous spinal direct current stimulation; TST, temporal summation threshold; VAS, Visual Analog Scale; W, women*.

## Results

### Identification and Selection of the Studies

A total of 279 records were identified. After removal of duplicates, 186 papers were screened through title and abstract and 9 papers were obtained for full-text screening. The reference lists of relevant papers were inspected for additional studies potentially missed in the databases search, but no significant papers were further added. Two authors (CZ, EM) independently evaluated the 9 papers selected for the full-text examination. Disagreement was solved by consensus between the two reviewers, therefore the advice of a third reviewer (ST) was not required.

**Figure 1:**
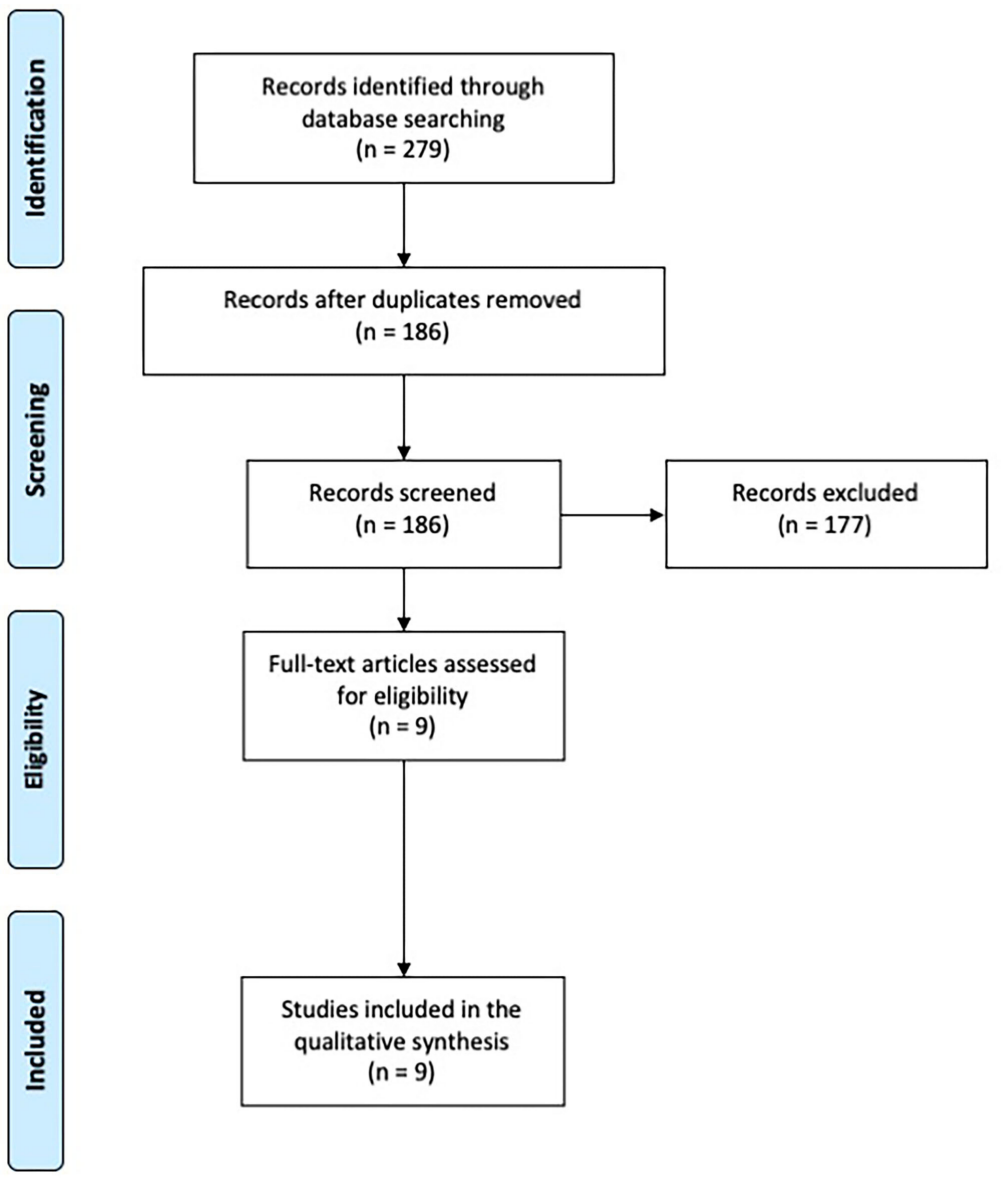
PRISMA diagram of the study (www.prisma-statement.org).

Nine studies fulfilled the criteria and were included in the systematic review (Figure 1).

### Description of the Included Studies

The included papers evaluated the efficacy of NIBSS on neuropathic or nociceptive pain in MS patients. Studies were grouped according to the NIBSS technique (i.e., tDCS, rTMS, tRNS, tsDCS).

### tDCS Studies

We found five studies that explored tDCS for the treatment of pain in MS (Mori et al., 2010; Ayache et al., 2016; Kasschau et al., 2016; Rudroff et al., 2019; Workman et al., 2020).

Mori et al. (2010) investigated whether anodal tDCS may be effective in reducing central drug-resistant chronic neuropathic pain in MS with a randomized, parallel, double blind, sham-controlled study. Nineteen patients with relapsing-remitting MS received a 5 day treatment with sham or real tDCS over M1 contralateral to the painful body region. Real tDCS resulted in significant reduction of pain and improvement of QoL in comparison to sham tDCS and the effects lasted up to 3 weeks after the stimulation period. The Authors hypothesized that pain reduction was the result of functional plastic changes in brain structures involved in the pathogenesis of chronic neuropathic pain (Mori et al., 2010).

Ayache et al. (2016) reported a prospective, randomized, cross-over, sham-controlled study to evaluate the effect of tDCS over the DLPFC in sixteen MS patients with a history of neuropathic pain since >3 months. The primary outcome was pain intensity, and secondary outcomes included mood, attention and fatigue. Patients received real or sham anodal tDCS blocks in a random order, each consisting of tDCS sessions in 3 consecutive days, separated by a 3 week washout period. Real tDCS yielded significant analgesic effects compared to sham, but no effects on mood, attention, or fatigue. The Authors suggested that analgesia might have occurred through specific modulation of the emotional pain network by tDCS over the left DLPFC (Ayache et al., 2016).

Since repeated tDCS sessions are needed to obtain a therapeutic effect, but for many MS patients visiting the clinic daily for the treatment is not feasible, Kasschau et al. (2016) performed a pilot study to test the feasibility and safety of a remotely supervised tDCS protocol for home delivery using a specially designed equipment and a telemedicine platform. Twenty MS (any subtype) patients with an extended range of disability (Expanded Disability Status Scale = 1–8) underwent 10 tDCS sessions over the left DLPFC, each lasting 20 min, across 2 weeks. Nineteen of them (95%) completed at least eight sessions, meeting the compliance criteria, while 17 (85%) completed the full 10 study sessions. Improvement of all secondary clinical outcomes (cognitive measures, pain, fatigue, mood) was reported. Despite the limitations of the study, i.e., lack of a control group, and patient economic compensation that might have increased the attendance, this telemedicine tDCS protocol suggests that access to tDCS can be expanded in MS patients (Kasschau et al., 2016).

Rudroff et al. (2019) reported a 52 year old man with a 13 year history of relapsing-remitting MS, moderate disability and central neuropathic pain, treated with anodal tDCS (20 min, 5 consecutive days) over the left M1. Pain scores improved and metabolism, assessed with [^18^F] fluorodeoxyglucose positron emission tomography, increased in both thalami, suggesting at a very preliminary stage that tDCS may induce functional changes in brain structures critical in the pathogenesis of neuropathic pain (Rudroff et al., 2019).

Workman et al. (2020) reported the results of a double blind, sham-controlled, randomized cross-over pilot study to investigate whether tDCS may improve the MS symptom cluster of pain, fatigue and depression. Six moderately disabled MS patients underwent two randomly ordered blocks of stimulation (real or sham tDCS), each block composed of five daily 20 min sessions, with the anode placed over the M1 representation of the more-affected leg and the cathode over the contralateral supraorbit. Real tDCS improved performance fatigability, perceived fatigue and pain but not depression in comparison to sham (Workman et al., 2020).

### rTMS Studies

We found two studies that explored rTMS in MS with pain as primary (Seada et al., 2013) or secondary outcome (Korzhova et al., 2019).

Seada et al. (2013) reported a randomized, parallel study that compared rTMS and low-level laser therapy for trigeminal neuralgia in thirty MS patients. Patients were randomly divided into rTMS group (age 46.6 ± 9.6) who received rTMS (10 Hz, 50 mA, 20 min) with the coil placed tangentially over the head of the patient contralateral to trigeminal pain and the laser group (age 48.8 ± 6.3) who received low-level laser therapy (15 mW helium-neon laser, 830 A wave length, 150–170 mw/cm^2^ laser beam density, 10 min). Both groups reported improvements, but no statistical comparison between the two groups was performed (Seada et al., 2013). Indeed, some methodological issues should be noted, such as poor description of rTMS targeting, absence of a sham group, unclear significance of outcome measures and some poorly reported data (e.g., the overall mean age was 56.4 ± 6.6 that is in contrast with the age of the two groups, see above).

Korzhova et al. (2019) performed a parallel, randomized controlled trial to compare the effects of two rTMS protocols, i.e., high frequency (20 Hz) and iTBS in comparison to a sham group on the level of spasticity (primary outcome) and associated symptoms, including pain that was a secondary outcome, in thirty-four secondary progressive MS patients. All patients underwent real (high frequency rTMS: twelve patients, iTBS: twelve patients) or sham rTMS (ten patients) once a day for 5 consecutive days for 2 weeks. Concurrently with rTMS, all patients received a course of 10 physical therapy sessions. Both high frequency rTMS and iTBS significantly reduced spasticity with some evidence favoring a longer-lasting effect of iTBS and a reduction of pain and fatigue to high frequency rTMS (Korzhova et al., 2019).

### Other NIBSS Studies

We found two studies (Table 3), one dealing with tRNS (Palm et al., 2016) and one with tsDCS (Berra et al., 2019).

Palm et al. (2016) explored the effect of tRNS over the left DLPFC on affective symptoms, attention, fatigue, and pain by exploring pain perception and attentional resources in a prospective randomized, cross-over, sham-controlled study of sixteen MS patients with neuropathic pain. Each patient randomly received two tRNS blocks (i.e., real, sham), each consisting of three consecutive 20 min daily sessions, separated by a 3 week washout interval. All patients were evaluated for pain, attention and mood and underwent a neurophysiological evaluation using pain related evoked potentials. Compared to sham, real tRNS showed a trend toward decreased N2-P2 amplitude of pain related evoked potentials and improvement of pain ratings, while attention performance and mood scales did not change (Palm et al., 2016).

Berra et al. (2019) explored whether anodal tsDCS could represent an effective, safe and well-tolerated treatment for neuropathic pain in MS in a double-blind sham-controlled, parallel design study involving thirty-three patients. Real tsDCS resulted in a significant improvement in neuropathic pain scores at the end of treatment that persisted 1 month later, but no changes in spasticity and fatigue. In a subgroup of patients, who underwent NWR and NWR TST, a non-significant trend toward an inhibition of NWR responses to real tsDCS was found, suggesting the effect of tsDCS was related to modulation of spinal nociception (Berra et al., 2019).

### Risk of Bias Assessment

**Figure 2:**
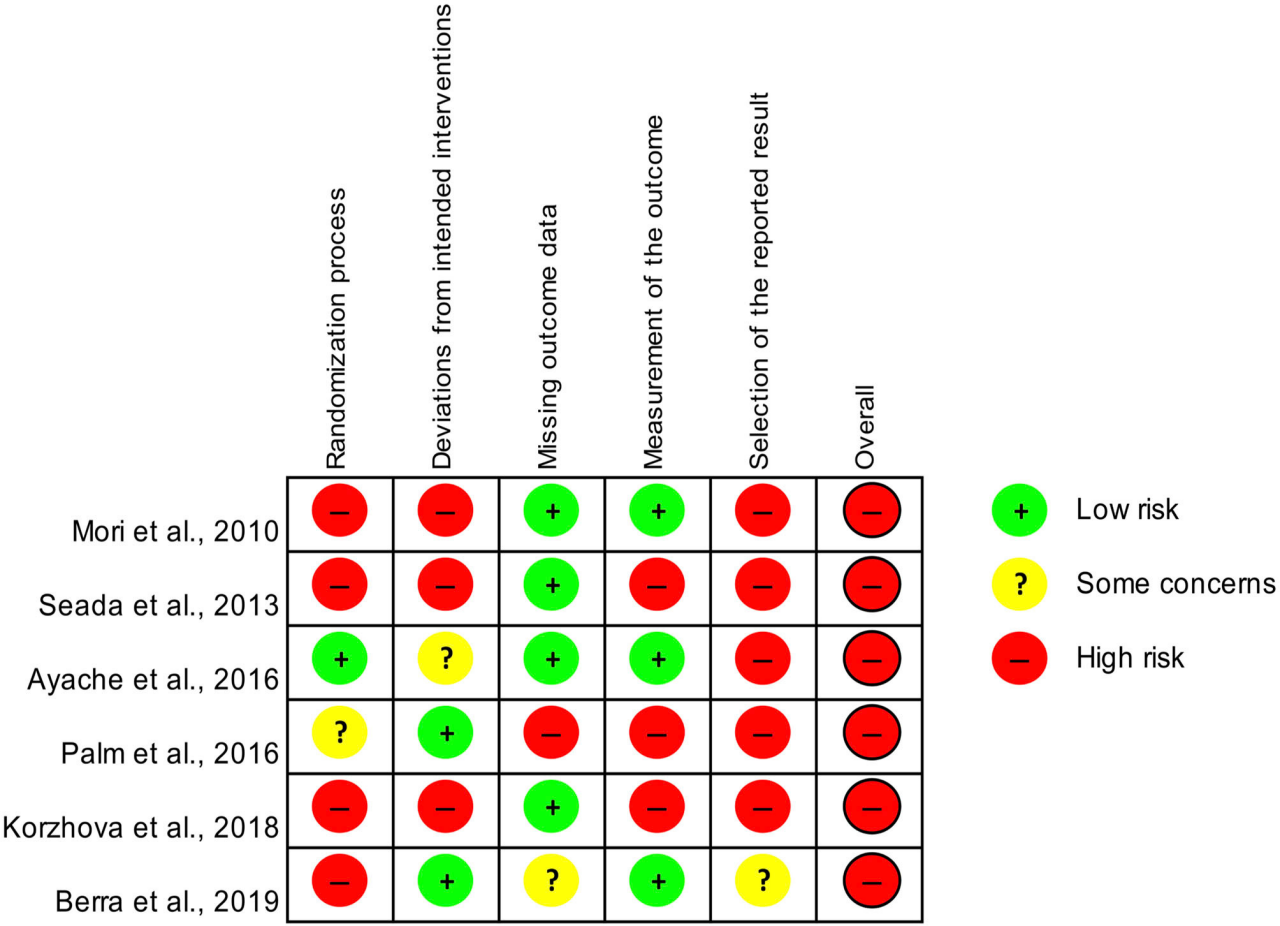
Assessment of the risk of bias for controlled studies included in the systematic review according to the RoB 2.0 tool.

Only controlled studies with samples of at least ten patients (Mori et al., 2010; Seada et al., 2013; Ayache et al., 2016; Palm et al., 2016; Berra et al., 2019; Korzhova et al., 2019) were assessed for the risk of bias according to the RoB 2.0 tool, which yielded an overall high risk for all of them (Figure 2).

## Discussion

The present systematic review, which was aimed to collect and report evidence on the role of NIBSS for the management of MS-related pain, yielded nine studies, of whom five on tDCS (Mori et al., 2010; Ayache et al., 2016; Kasschau et al., 2016; Rudroff et al., 2019; Workman et al., 2020), two on rTMS (Seada et al., 2013; Korzhova et al., 2019), one on tRNS (Palm et al., 2016), and one on tsDCS (Berra et al., 2019).

Four studies targeted M1, all of them using excitatory protocols, i.e., anodal tDCS in three of them (Mori et al., 2010; Rudroff et al., 2019; Workman et al., 2020), and high-frequency rTMS in another one (Korzhova et al., 2019), with the targeted side being the one contralateral to the most affected limbs in three of them (Mori et al., 2010; Korzhova et al., 2019; Workman et al., 2020), and the left side in another one (Rudroff et al., 2019). Three studies targeted the left DLPFC with excitatory protocols, i.e., anodal tDCS in two of them (Ayache et al., 2016; Kasschau et al., 2016) and tRNS in another one (Palm et al., 2016). One study targeted the spinal cord with anodal tsDCS (Berra et al., 2019) and the brain target was not specified in one study (Seada et al., 2013).

Seven studies had a randomized design, either double-blind (Mori et al., 2010; Ayache et al., 2016; Palm et al., 2016; Berra et al., 2019; Workman et al., 2020), single-blind (Korzhova et al., 2019), or with blinding not specified (Seada et al., 2013), one had an open design (Kasschau et al., 2016), and another one was a single case report (Rudroff et al., 2019). Sham NIBSS was the control group in six of the seven randomized studies (Mori et al., 2010; Ayache et al., 2016; Palm et al., 2016; Berra et al., 2019; Korzhova et al., 2019; Workman et al., 2020), while low-level laser therapy was used as control condition in one study (Seada et al., 2013).

Only three studies reported a follow-up that ranged from 4 to 12 weeks (Mori et al., 2010; Berra et al., 2019; Korzhova et al., 2019).

Apart from the single case report, the sample size ranged from 6 to 34 with a total of 175 patients (women: 92, men: 53; gender not specified: 30) included in the nine studies we found; among them there were 58 relapsing-remitting patients, 78 secondary progressive patients, 9 primary progressive patients, while MS type was not specified in 30 patients.

Pain was the primary outcome in seven of the included studies (Mori et al., 2010; Seada et al., 2013; Ayache et al., 2016; Palm et al., 2016; Berra et al., 2019; Rudroff et al., 2019; Workman et al., 2020) and secondary outcome in the other two studies, one being on feasibility of a telemedicine tDCS protocol (Kasschau et al., 2016), and the other one having spasticity as the primary outcome (Korzhova et al., 2019). The type of MS-related pain addressed in the study, either as primary or secondary outcome measures was neuropathic pain in seven studies (Mori et al., 2010; Seada et al., 2013; Ayache et al., 2016; Palm et al., 2016; Berra et al., 2019; Rudroff et al., 2019; Workman et al., 2020), more specifically central neuropathic pain in one of them (Mori et al., 2010) and trigeminal neuralgia in another one (Seada et al., 2013), while pain type was not specified in one study (Kasschau et al., 2016), and another study was focused on spasticity-related pain (Korzhova et al., 2019).

One or more of the other symptoms belonging to the symptoms cluster associated with pain in MS, i.e., fatigue, depression and cognitive complaints (Penner et al., 2007; Trojan et al., 2007; Harrison et al., 2015; Marck et al., 2017), and QoL were assessed as primary or secondary outcomes in six studies (Mori et al., 2010; Ayache et al., 2016; Kasschau et al., 2016; Palm et al., 2016; Berra et al., 2019; Korzhova et al., 2019).

The variability of NIBSS techniques, central nervous system targets, study designs including sham control and blinding, patient populations, outcomes, and the presence of follow-up data in a minority of the studies included did not allow a meta-analysis of the findings. However, the results of all the included studies converge in showing a significant improvement in pain after active NIBSS with less consistent effects on the other symptoms of the pain-related cluster and QoL (Mori et al., 2010; Seada et al., 2013; Ayache et al., 2016; Kasschau et al., 2016; Palm et al., 2016; Berra et al., 2019; Korzhova et al., 2019; Rudroff et al., 2019; Workman et al., 2020).

Most studies used validated scales to measure pain, such as the Visual Analog Scale, the Numerical Rating Scale, the Short Form McGill Pain Questionnaire, the Brief Pain Inventory or similar outcomes (Jensen and Karoly, 2001). However, despite most of the reports addressed neuropathic pain, only one study (Berra et al., 2019) used an outcome measure that was specific for this type of pain, i.e., the Neuropathic Pain Symptom Inventory (Bouhassira et al., 2004), which can be used to characterize subgroups of neuropathic pain patients and verify whether they respond differentially to a therapeutic intervention (Magrinelli et al., 2013). Moreover, because of the complexity of pain experience, and the coexistence of psychiatric and cognitive symptoms (Chiaravalloti and De Luca, 2008), MS patients may have difficulty in reporting their experience through a single intensity scale (Amatya et al., 2018), but only one study used a multidimensional pain scale, i.e., the Brief Pain Inventory (Ayache et al., 2016).

Some studies explored the MS symptom cluster related to pain. Depression, anxiety, mood, and affect were explored in five studies (Mori et al., 2010; Ayache et al., 2016; Kasschau et al., 2016; Palm et al., 2016; Workman et al., 2020), but found to improve to NIBSS only in one of them whose design was unblinded and open (Kasschau et al., 2016) and thus prone to placebo effect. The absence of changes in mood outcomes in the studies targeting the DLPFC (Ayache et al., 2016; Palm et al., 2016), might be due to the short stimulation period, since the effect on mood to DLPFC non-invasive stimulation is known to be dose-dependent (Palm et al., 2016).

Fatigue was an outcome measure in six studies (Ayache et al., 2016; Kasschau et al., 2016; Palm et al., 2016; Berra et al., 2019; Korzhova et al., 2019; Workman et al., 2020), with one of them performing a fatigability test with isokinetic leg strength in addition to subjective measures (Workman et al., 2020). Apart from the open feasibility study that found an unspecific improvement of all outcomes (Kasschau et al., 2016), the other studies converge in showing improvement of fatigue and fatigability to M1 excitatory NIBSS (Korzhova et al., 2019; Workman et al., 2020), but no effect to either targeting DLPFC (Ayache et al., 2016; Palm et al., 2016), or the spinal cord (Berra et al., 2019), thus suggesting that M1 may represent an interesting target for this MS symptom, which is very bothersome, may heavily impact on QoL and functioning, and has no established treatment (Miller and Soundy, 2017). However, it is worth noting that M1 reports applied 5 (Workman et al., 2020) and 10 sessions (Korzhova et al., 2019), respectively, whereas DLPFC studies (Ayache et al., 2016; Palm et al., 2016) applied only 3 sessions. Studies that targeted primary fatigue in MS documented significant effects by stimulating the DLPFC for ≥5 sessions and suggest that targeting the MS fatigue loop with 5 or more NIBSS sessions could improve the symptom (Ayache and Chalah, 2018). In MS patients with pain, fatigue is probably secondary to pain rather than representing primary fatigue, and future studies should better explore this topic.

Two studies explored the effect of NIBSS on spasticity (Berra et al., 2019; Korzhova et al., 2019) and showed improvement of this outcome to high-frequency rTMS over M1 (Korzhova et al., 2019), but not to anodal tsDCS (Berra et al., 2019), offering some ground to future studies aimed to explore excitatory NIBBS over M1 as a therapeutic strategy in MS-related spasticity.

Three studies explored attentional changes to left DLPFC anodal tDCS (Ayache et al., 2016; Kasschau et al., 2016) and tRNS (Palm et al., 2016). Only one of them (Kasschau et al., 2016) reported improvement of attentional outcomes, but the open design of the study, absence of blinding, and coexisting treatment with web-based cognitive rehabilitation might have represented potential bias factors. The left DLPFC might not represent the best target for this cognitive domain, which is frequently impaired in MS (Chiaravalloti and De Luca, 2008), as anodal tDCS over the right posterior parietal cortex was reported to be more effective on attentional measures than the left DLPFC (Roy et al., 2015).

QoL was explored in one study only, with no evidence of efficacy of NIBSS (Mori et al., 2010), probably because this outcome is likely to improve in response to change of a symptom cluster instead of a single symptom (Ehde et al., 2003; Svendsen et al., 2003, 2005; Kalia and O'Connor, 2005).

Three studies presented follow-up data and were consistent in showing that NIBSS effects outlasted the period of stimulation; in particular, pain reduction lasted up to 1 month after the end of NIBSS treatment (Mori et al., 2010; Berra et al., 2019; Korzhova et al., 2019).

Some interesting pieces of information can be derived from the instrumental outcomes reported in some of the studies we collected. Rudroff et al. (2019) documented increased [^18^F] fluorodeoxyglucose positron emission tomography uptake in the thalamus after anodal tDCS and suggested that NIBSS may modulate sensory discriminative and affective-motivational pain pathways. However, this finding was derived from a single case report and should be replicated in larger patient populations. Moreover, validated and clinically reliable neuroimaging markers of MS-related pain are still lacking (Seixas et al., 2014). Palm et al. (2016) reported reduced amplitude of the N2-P2 component of pain related evoked potentials. This finding might be related to a change in cortical processing of pain, but should be taken with care, because of the uncertainties on the fibers stimulated by the electrode they used (Perchet et al., 2012) and the presence of saliency and habituation effects that may represent bias factors for the interpretation of the significance of the cortical components to pain stimuli (Iannetti et al., 2008). Berra et al. (2019) reported a trend for a change in NWR in parallel to neuropathic pain improvement to tsDCS. NWR is a reliable neurophysiological tool for the assessment of the spinal and supraspinal mechanisms of pain processing but is sensitive to physiological changes and to some drugs (Sandrini et al., 2005) and not widely used in the clinical setting.

M1 and the DLPFC were the two most common NIBSS sites for the treatment of MS-related pain in the studies we reviewed. M1 stimulation is supposed to induce analgesic effects trough an antidromic top-down modulation of thalamo-cortical pathways (Nguyen et al., 2011). The DLPFC plays a pivotal role in pain processing, as well as cognitive and emotional pain-related behaviors, and its stimulation may act through a descending modulation of opioidergic pathways and in the affective and attentional aspects of pain (Seminowicz and Moayedi, 2017). However, the underlying brain networks that mediate pain relief to these brain targets are only partially understood, and they may be partially disrupted in patients with MS.

In conclusion, the results of the studies included in this systematic review indicate overall a positive effect of various NIBSS techniques on pain and some related symptoms in patients with MS. These results are promising but far from being conclusive, because of the small sample size in the included studies, the variability in NIBSS technique, targeted area, patient population, outcomes, the absence of follow-up for many of the studies, and the overall high risk of bias. It is worth noting that the assessment of the risk of bias in our study differs from that in a recent Cochrane review focused on the management of chronic pain in MS patients (Amatya et al., 2018), because of the different risk of bias tools used in the two studies. Excitatory NIBSS over M1, the left DLPFC and the spinal cord appear to be the most promising protocols to be used in future larger therapeutic studies for MS-related pain.

Open questions include the use of neurophysiological or neuroimaging surrogate outcome measures and the stratification of patients according to the clinical profiles and underlying pathogenetic mechanisms (Magrinelli et al., 2013). Future studies should explore whether NIBSS protocols associated to pharmacological treatment, neurorehabilitation, or psychotherapy (Arewasikporn et al., 2018) may be more effective than NIBSS alone on pain, related symptoms and/or QoL.

The duration of the effect to NIBSS is another key question. Unlike other clinical conditions such as depression, there is still no consensus regarding the treatment of MS related pain with maintenance sessions of NIBBS beyond the normal treatment duration. Studies on other neuropathic pain conditions suggest that the analgesic effect to rTMS of M1 is favored by longer session duration and serial treatment, i.e., greater number of sessions (Lefaucheur et al., 2020). Additional studies are thus needed to address this important question.

Regarding the last point, the use of telemedicine NIBSS techniques may be promising (Kasschau et al., 2016), but results are still contradictory, in that a randomized controlled pilot study documented that patient-delivered tDCS was not effective on mixed types of neuropathic pain in prior responders to rTMS (O'Neill et al., 2018). Telemedicine could also lead to advantages for designing future NIBBS clinical trials to test more appropriate stimulation parameters, treatment duration and follow-ups. The remote provision of NIBSS (e.g., tDCS), safely administered at home may be an interesting option to provide accessible maintenance protocol treatments, to explore the effects of NIBSS in an ecological context, and to overcome the limitations of not-portable NIBBS devices. The use of telemedicine could also be helpful for research purposes, allowing for a better control of experimental variables and thus increasing the reproducibility of studies' findings.

## Author Contributions

The study has been designed by CZ, EM, RD, CT, GS, and ST. Data have been gathered by CZ and EM, under the supervision of ST. Data have been analyzed by CZ and EM. The manuscript has been drafted by CZ, EM, and ST. RD, CT, and GS revised the manuscript for important intellectual content. All authors approved the final version of the manuscript.

## Conflict of Interest

The authors declare that the research was conducted in the absence of any commercial or financial relationships that could be construed as a potential conflict of interest.
